# First evidence of Retzius-sparing robot-assisted radical prostatectomy with the Versius robotic surgical system

**DOI:** 10.3389/fonc.2025.1655088

**Published:** 2025-09-08

**Authors:** Erika Palagonia, Stefano Tappero, Elio Mazzone, Francesco Chierigo, Carlo Buratto, Alberto Caviglia, Michele Barbieri, Ofir Maltzman, Valerio Cellini, Giancarlo Napoli, Alberto Olivero, Elena Strada, Dario Di Trapani, Giovanni Petralia, Silvia Secco, Aldo Massimo Bocciardi, Antonio Galfano, Paolo Dell’Oglio

**Affiliations:** Department of Urology, ASST Grande Ospedale Metropolitano Niguarda, Milan, Italy

**Keywords:** Retzius-sparing, prostatectomy, Versius, robotic surgery, prostate cancer

## Abstract

We describe the first experience of Retzius-sparing robot-assisted radical prostatectomy (RS-RARP) performed with the Versius robotic system (CMR Surgical, Cambridge, UK). Five patients underwent RS-RARP at ASST Grande Ospedale Metropolitano Niguarda (Milan, Italy) between May 2023 and December 2023. All procedures were completed with no complications or need for conversion. No arms collision or technical failure of the system was recorded. The median operative time was 244 minutes (interquartile range [IQR]: 190-300). Median length of stay was three days. Our initial experience suggests that RS-RARP with the Versius robotic system is a feasible procedure. This novel platform provides a flexible system environment that can be easily tailored to all types of surgery. However, some arrangements are still necessary, warranting future investigations in larger series.

## Introduction

Novel robotic surgical platforms have recently emerged as promising alternatives to the Intuitive systems, that hold the field for approximately twenty years. Surgical procedures might need technical refinements according to different robotic systems. In consequence, reproducibility, safety and feasibility must be carefully assessed before widespread use of a new robotic platform in daily practice.

This might also apply to Retzius-sparing robot-assisted radical prostatectomy (RS-RARP) performed with the novel Versius robotic platform (CMR Surgical, Cambridge, UK). RS-RARP represents a valuable minimally invasive surgical treatment for localised prostate cancer (PCa) (1). Its adoption has been fully standardized and described with the usage of DaVinci Si and Xi systems (Intuitive Surgical, Sunnyvale, CA, USA). This concerned all clinical scenarios, also including high-risk PCa patients (2), presence of bulky median lobe, large prostate glands (3), and history of previous surgery for benign prostate obstruction (4).

Versius is a modular robotic system including the following: a) a surgeon open console with ergonomic hand controllers with energy button, no pedal control; b) a surgeon head-up display with a three-dimensional screen and the possibility for the surgeon to control the robot either sitting or standing; c) an optic bedside unit (BSU); d) up to five movable operative BSUs for different instruments (5). Each component has a wristed joint that mimics the human articulations and provides seven degrees of freedom at the instrument tip for an overall 720° rotation. Each instrument has length of 30 cm, while instruments diameters vary from 6.8mm to 10mm (endoscope), and they can be used up to 20 times depending on the instrument type. The modest size of each module and its mobility provide a flexible system environment that can be easily tailored to all types of surgery. Due to the short instrument length, the Versius system, in the urologic field, has mostly been used for renal surgery (6, 7). Nonetheless, first data on its adoption in standard anterior RARP have been recently recorded (8). To date, only one case of RS-RARP with Versius system has been described. However, this exclusively concerned cadaveric models (9), whether no data on human PCa patients are available. In the current study, we present preliminary data of RS-RARP without lymph node dissection performed with the novel Versius robotic system at the ASST Grande Ospedale Metropolitano Niguarda of Milan (Italy). Specifically, the description of the operative room setting, trocar and BSU placement, as well as the assessment of safety and reproducibility of the technique are provided in detail.

## Patients and methods

**Figure 1:**
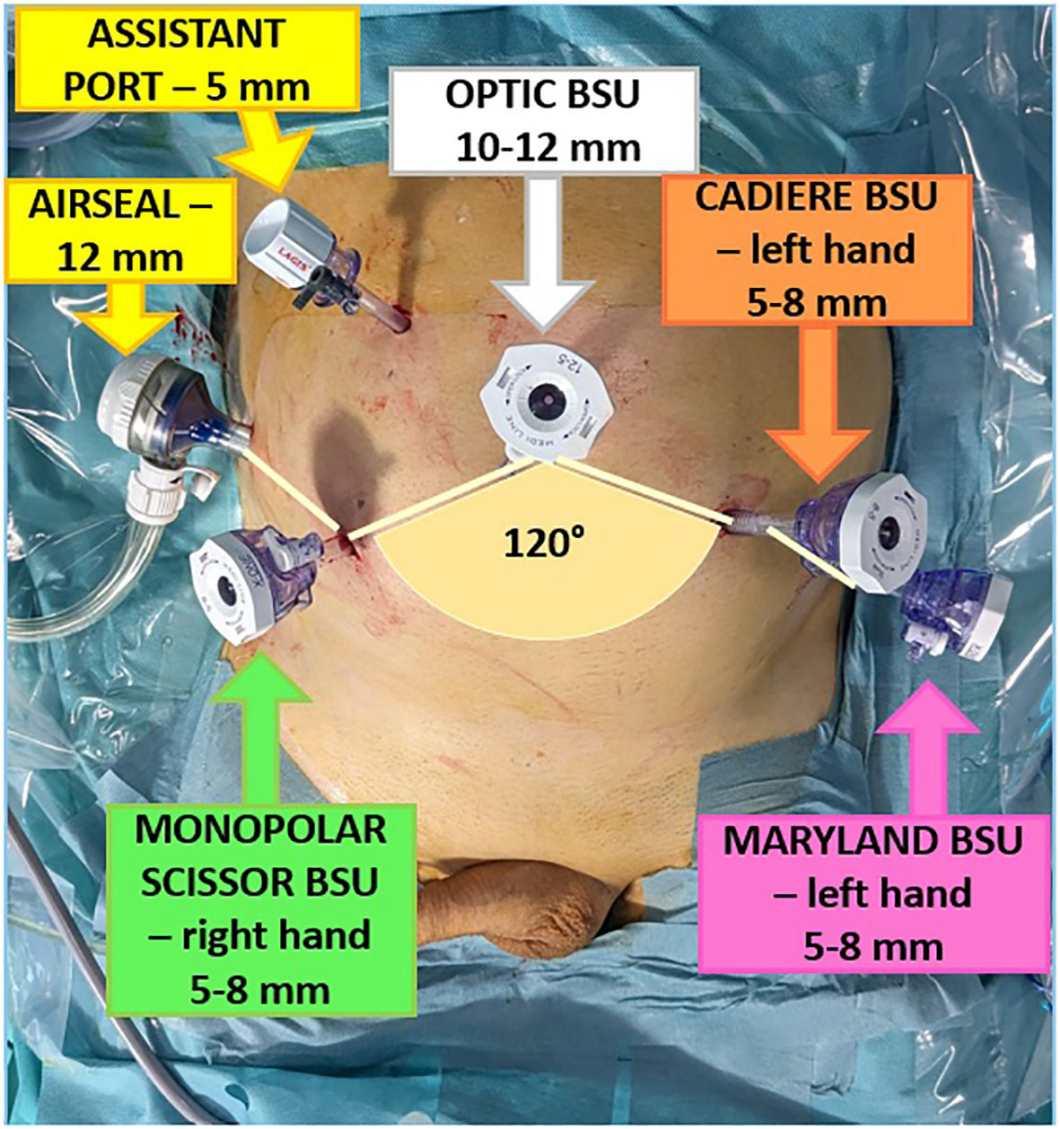
Trocars configuration.

**Figure 2:**
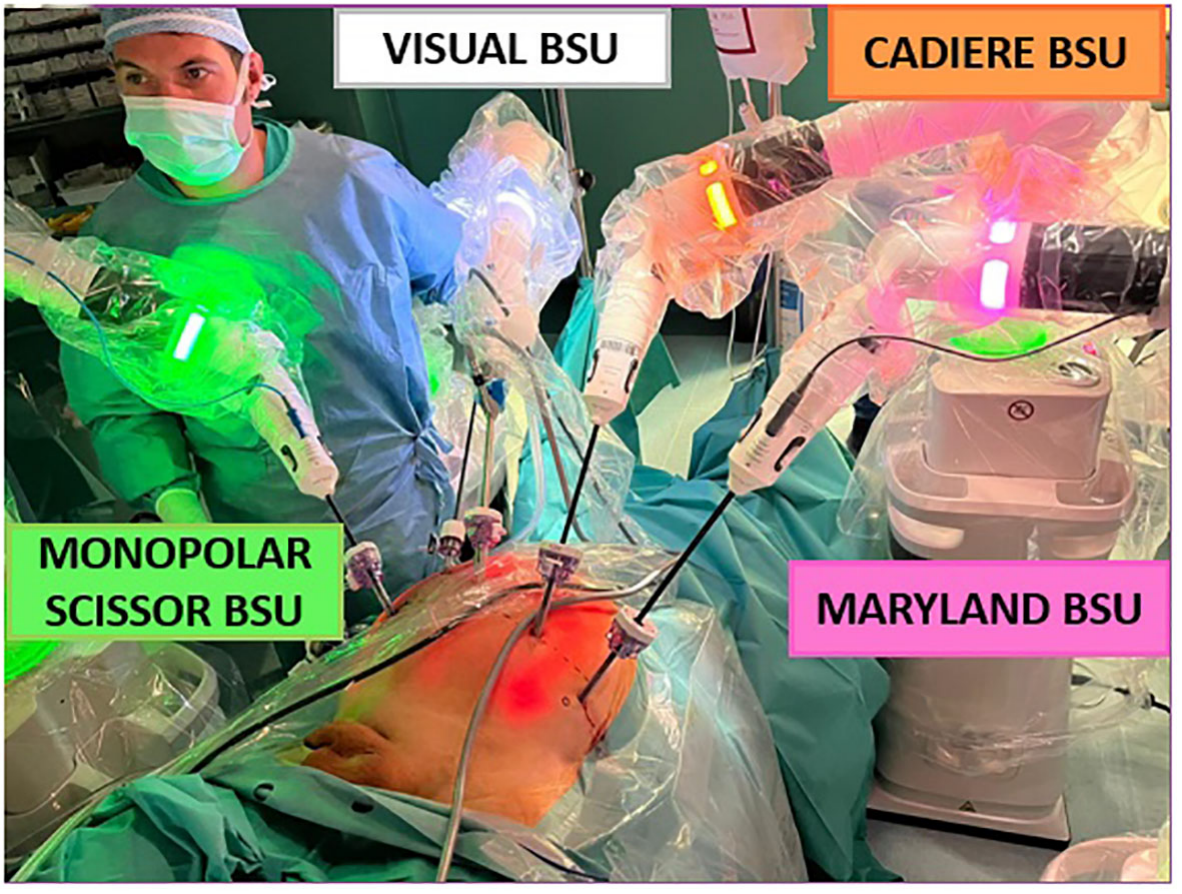
Bedside units’ configuration.

First, the current study aimed to report the surgical setting of Versius system to perform RS-RARP. The secondary endpoint was to assess the feasibility of RS-RARP with this novel robotic platform and to report perioperative outcomes. Pre, intra and postoperative data were collected. Categorical and continuous variables were reported as frequencies with proportions, and medians with interquartile ranges (IQR), respectively. All statistical analyses were performed using R software Version 4.1.3 (R Foundation for Statistical Computing, Vienna, Austria). All surgeries were completed by two expert robotic surgeons (DDT, ES). The surgical team underwent official theorical and technical training on dry and wet laboratory (i.e., human cadavers) at IRCAD (Strasbourg, France), with a specific focus on RS-RARP with lymph node dissection and partial nephrectomy. This study was conducted in accordance with the principles of Good Clinical Practice and the Declaration of Helsinki, all patients signed an informed consent, and the study was approved by the internal ethic committee of the hospital. Before the first in-human procedure, the best operative setup and port placement was tested in dry and wet lab, as well as on human cadavers, during the training at IRCAD. Patients were placed in a 30° Trendelenburg position and a 12 mmHg intra-abdominal pressure was used. One 12-mm endoscope port was placed on the midline approximately 2 cm above the umbilicus (**Figure 1**). The 8-mm trocar for the right robotic (R1) and left robotic (R2) arm were positioned at least 8 cm lateral and 4 cm caudal, drawing an angle between 100° and 120° with the endoscope port. The 8-mm trocar for the other left robotic arm (R3) was placed 8 cm lateral to the R2 port, following an imaginary lunar line as in laparoscopic prostatectomy. A first 5-mm assistant port (A1) was positioned between endoscope port and R1 port, at least 3 cm cranially and 4 cm lateral or medial, and a second 12-mm assistant port (A2) was placed on a transversal line from the camera port, 4–5 cm cranial to the right iliac crest. First of all, it is necessary to establish the orientation and height of each module before activation and positioning at the patient’s bedside. The unit arm must be opened and moved toward the patient side to start the calibration process on the target area. The surgical access calibration mode starts when the instrument is attached to the robotic arm and inserted inside the patient’s cavity for approximately 2 cm, then the calibration fulcrum is identified. These steps are necessary to avoid force transmission to the trocar on the patient’s abdomen, since, unlike other robotics systems, Versius is not docked to the trocars. Subsequently, it is possible to move the V-Wrist arm forming a circular arc clockwise and counter-clockwise, until the calibration is finished. These steps are mandatory for all the instruments, in order to proceed with the BSU arms for instruments on the right-hand side (scissors and needle driver) and subsequently the two BSUs on the left side. The last arm positioned is the optic BSU that is normally placed above the head towards the right-hand side, leaving space for the table assistant (**Figure 2**).

## Results

**Figure 3:**
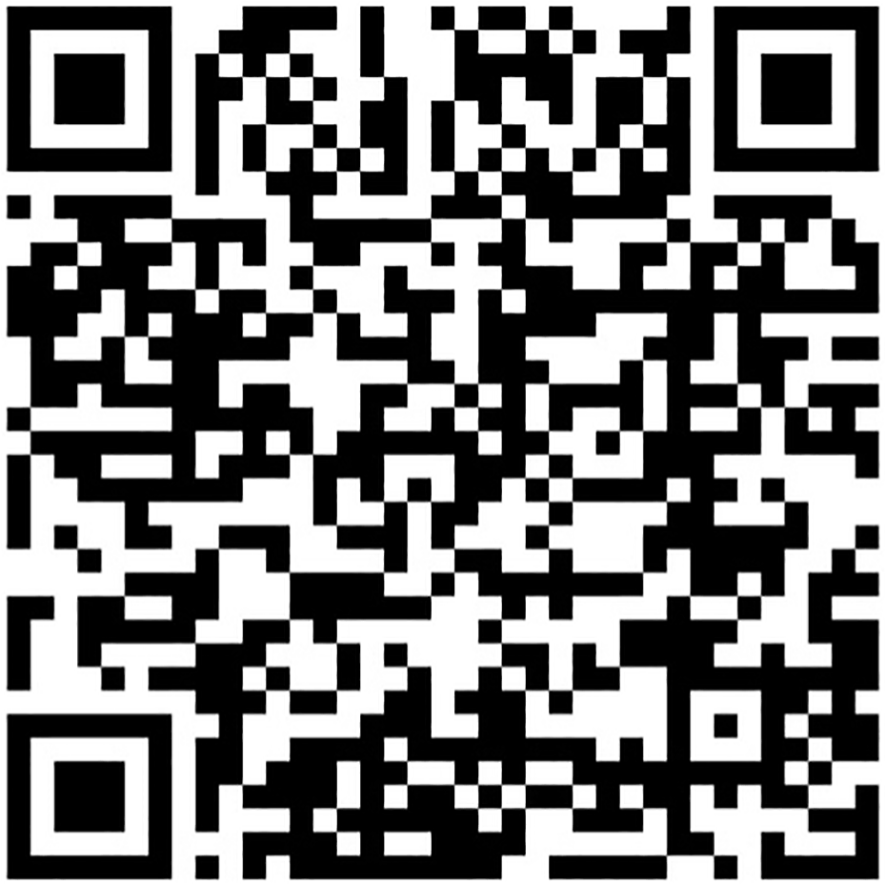
Video: step by step.

**Table 1** shows patients characteristics and procedural details. Five patients underwent RS-RARP at ASST Grande Ospedale Metropolitano Niguarda (Milan, Italy) between May 2023 and December 2023. Median age was 63 years old. Median body mass index was 27,1 kg/m^2^. All patients had a diagnosis of organ-confined prostate cancer ISUP [International Society of Urological Pathology] 1, and prostate-specific antigen ≤10 ng/ml. According to the Memorial Sloan Kettering and Briganti nomograms the pelvic lymph nodes dissection was not required. The median docking time was 43 minutes, and the median console time was 244 minutes. All the surgical steps were completed without critical problems (**Figure 3**). No collisions between the instruments were recorded. No complications were recorded during and after surgery. In the first case, the transurethral catheter was placed instead of suprapubic tube due to a bulky median lobe that hampered complete bladder neck preservation. **Table 2** shows post-operative details. All five patients were discharged on postoperative day three. Two patients had a positive focal surgical margin (i.e., extension <3 mm), and one patient had a locally advanced disease at the final pathology (i.e., pT3a). After one week catheter was removed and complete continence (daytime use of no safety pads/a single safety pad) was recorded for all patients.

**Table 1:** Patients and procedure details.

Patient number	Patient 1	Patient 2	Patient 3	Patient 4	Patient 5
Age (yr)	63	51	64	67	70
Body mass index	24,2	28,4	25,1	28,7	29,3
Preoperative PSA (ng/ml)	5,46	1,5	7,22	7,36	5,1
Biopsy ISUP grade group	1	1	1	1	1
Prostate volume in preoperative MRI (cm^3^)	111	26	42	76	77
T stage on preoperative MRI	T1c	T1c	T1c	T1c	T1c
Total operative time (min)	350	255	260	360	275
BSUs position time (min)	50	37	45	50	35
Console time (min)	290	190	200	300	240
Estimated blood loss (ml)	200	100	120	150	100
Bladder drain (TC: transurethral catheter, SC: suprapubic catheter)	TC	SC	SC	SC	SC

PSA, prostatic specific antigen; ISUP, International Society of Urological Pathology; MRI, magnetic resonance imaging; BSU, bed side unit.

**Table 2:** Post-operative details.

Post-operative data	Median (IQR
Length of stay (d)	3 (3 – 3)
Suprapubic or transurethral catheter removal, (d)	7 (7-7)
ISUP grade group at final pathology	n (%)
1	1 (20)
2	3 (60)
4	1 (20)
pT stage	n (%)
pT2	4 (80)
pT3a	1 (20)
Positive surgical margins	n (%)
No	2 (40)
Focal (<3 mm)	2 (40)
Yes	1 (20)
Continence recovery	n (%)
1 week after surgery	5 (100)

ISUP, International Society of Urological Pathology.

## Discussion

We demonstrated the feasibility and safety of RS-RARP using the novel Versius system. We identified an ideal configuration for this new robotic platform in a setup that resulted in no intraoperative complication or system failure. One of the main advantages of Versius system lies in its modularity. Specifically, robotic surgical systems typically fix robotic arms to a single cart, and in one system the robot is located over the patient. These designs can present challenges with maneuverability, restrict physical space, and inhibit access to the patient. On the contrary, Versius module arms have eight articulating joints, and the instruments are wristed, providing seven degrees of freedom for precise and stable movements while operating. Additionally, the instrument and visualization BSUs are mobile and practically sized for ease of transport, thus removing the need for a specialized operating room. Moreover, the elbow of the instrument arm can be moved by surgical personnel while Versius is in surgical mode facilitating better access to the patient. Another interesting feature is the hand controller, which was designed ergonomically with surgeon input to optimize comfort. Specifically, all Versius controls are placed on the handles, removing the need for foot pedal controls. Moreover, tailored hand scaling of the articulated wrists via a multiplier facilitates surgery, especially during stitching. Additionally, by the use of the joysticks placed on the handles it is possible to move the camera and the robotic instruments simultaneously. Finally, the surgeon console has an open (i.e., non-immersive) design that allows the surgeon to maintain communication with their team during surgery and its height is adjustable, providing the option to sit or stand while operating. This feature might allow laparoscopic surgeons, who are accustomed to work while standing, a smoother transition to robotic surgery, and might be also useful for training and teaching purposes, as observers and trainees might follow the operation by the side of the surgeon using 3D vision. Of note, the endoscopic camera provides an 81.1°field of view with a 300 mm working length. With respect to possible disadvantages, Versius system does not allow to rotate the endoscope by use of the robotic handles, and the endoscope needs to be manually detached from the BSU, rotated and re-attached again. This might be limiting in RS-RARP, where a 30° endoscope needs to be rotated during the operation to allow correct visualization. Another limitation concerning vision is the fact that the assistant screen has dark spots compared to the console monitor, requiring frequent movements of the camera to have the target area in the middle of the screen, and the lack of integration of external tools such as fluorescence detector or ultrasound scans. Moreover, it can be argued that 30cm instrument length might be too short for surgeries of the lower pelvis, especially in tall patients or those with high BMI. Performing a procedure with a new complex surgical device is expected to be associated with a slower time of surgery as the teams gain familiarity with the system and instrumentation. For example, one could argue that the docking process for multiple arm carts might be time-consuming. In our experience, the new docking method of Versius system translated into longer docking times (43 minutes vs an average 10–20 minutes with DaVinci and Hugo-RAS systems). Operative time was slightly longer compared to our experience with DaVinci system, but it was comparable to our initial experience with Hugo-RAS system. As the hospital team becomes increasingly familiar and confident, we believe that operative times will decrease. However, as safety is of paramount importance, conclusions drawn from metrics such as operative times should be moderated and not taken as an authoritative measure of patient outcome. Last but not least, regarding the specific results of the current series, our rate of positive margins was indeed higher compared to other series using Versius (10). In this context, it might be hypothesized that RS-RARP might require slightly longer learning curve compared to standard anterior approaches, but further studies are needed to confirm this point.

This study is not devoid of limitations. First, despite prospective data collection, the number of procedures performed is limited. As the current series represents our initial experience, easier cases were selected, while we performed more challenging surgeries with DaVinci Xi. However, we are currently broadening the indications to more challenging cases. Second, the procedures were performed by surgeons with remarkable robotic experience, thus making results not reproducible by surgeons approaching robotic surgery for the first time with Versius system. Finally, despite reporting of oncological and functional was beyond the scope of this article, these results will be of utmost importance to understand whether Versius system represents a true alternative to Intuitive platforms. Another limitation might be identified in not following IDEAL Guidelines. Within these limitations, our study provides insights on the surgical setting and the feasibility of RS-RARP with Versius, that may be crucial to make the first steps with this novel robotic platform.

## Conclusions

Our initial experience suggests that RS-RARP can safely be reproduced with the novel Versius robotic system. However, formal training, careful surgical planning and accurate patient selection are key while introducing a new robotic system into the routinary practice. Larger series, longer follow up periods and proper comparisons with alternative robotic platforms will invariably provide additional remarkable clues about the reliability of RS-RARP performed with Versius.

## Data Availability

The datasets presented in this article are not readily available because institutional dataset. Requests to access the datasets should be directed to paolo.delloglio@ospedaleniguarda.it.
